# Establishing Imaging Biomarkers of Host Immune System Efficacy during Glioblastoma Therapy Response: Challenges, Obstacles and Future Perspectives

**DOI:** 10.3390/metabo12030243

**Published:** 2022-03-14

**Authors:** Ana Paula Candiota, Carles Arús

**Affiliations:** 1 Centro de Investigación Biomédica en Red en Bioingeniería, Biomateriales y Nanomedicina (CIBER-BBN), Cerdanyola del Vallès, 08193 Barcelona, Spain; anapaula.candiota@uab.cat; 2 Departament de Bioquímica i Biologia Molecular, Unitat de Bioquímica de Biociències, Edifici Cs, Universitat Autònoma de Barcelona, Cerdanyola del Vallès, 08193 Barcelona, Spain; 3 Institut de Biotecnologia i de Biomedicina (IBB), Universitat Autònoma de Barcelona, Cerdanyola del Vallès, 08193 Barcelona, Spain

**Keywords:** glioblastoma, cancer immune cycle, immunotherapy, host immune system, magnetic resonance spectroscopic imaging, metabolomics, imaging biomarker

## Abstract

This hypothesis proposal addresses three major questions: (1) Why do we need imaging biomarkers for assessing the efficacy of immune system participation in glioblastoma therapy response? (2) Why are they not available yet? and (3) How can we produce them? We summarize the literature data supporting the claim that the immune system is behind the efficacy of most successful glioblastoma therapies but, unfortunately, there are no current short-term imaging biomarkers of its activity. We also discuss how using an immunocompetent murine model of glioblastoma, allowing the cure of mice and the generation of immune memory, provides a suitable framework for glioblastoma therapy response biomarker studies. Both magnetic resonance imaging and magnetic resonance-based metabolomic data (i.e., magnetic resonance spectroscopic imaging) can provide non-invasive assessments of such a system. A predictor based in nosological images, generated from magnetic resonance spectroscopic imaging analyses and their oscillatory patterns, should be translational to clinics. We also review hurdles that may explain why such an oscillatory biomarker was not reported in previous imaging glioblastoma work. Single shot explorations that neglect short-term oscillatory behavior derived from immune system attack on tumors may mislead actual response extent detection. Finally, we consider improvements required to properly predict immune system-mediated early response (1–2 weeks) to therapy. The sensible use of improved biomarkers may enable translatable evidence-based therapeutic protocols, with the possibility of extending preclinical results to human patients.

## 1. Introduction

Glioblastoma (GB) is the most aggressive glial brain tumor type and cannot yet be cured in patients. The median overall survival (OS) after standard therapy is below 15 months [1,2], with a 10-year survival rate of only 0.71% [3]. The standard therapeutic protocol for GB patients is maximal surgical resection (whenever possible) and concomitant radiotherapy and temozolomide (TMZ) chemotherapy, followed by adjuvant TMZ. Still, the proposal that the efficacy of major therapies against GB requires the participation of the host immune system (IS) is gaining acceptance [4,5,6,7]. In this sense, the overall process is mediated by immune system recruitment by “damage associated molecular patterns” (DAMP) exposed or released by tumor cells during immunogenic cell death (ICD) processes, triggered by therapy with the participation of both the innate and adaptive arms of the IS in killing tumor cells [8,9,10,11]. Moreover, independently of the therapeutic strategy triggering its participation, there should be a common IS-driven response pattern, provided the treatment is at least transiently effective in damaging cancer cells.

This paper provides a hypothesis proposal for dealing with three questions related to the non-invasive assessment of immune system action in GB and its current status: (1) Why do we need imaging biomarkers for assessing the efficacy of immune system participation in GB therapy response? (2) Why are they not available yet? and (3) How can we produce them?

## 2. Hypothesis Proposal

### 2.1. Why Do We Need Imaging Biomarkers for Assessing the Efficacy of Immune System Participation in GB Therapy Response?

Ideally, clinicians would like to maximize the effectiveness of therapy used with GB patients, while minimizing unwanted systemic effects that may lead to therapy being halted or discontinued. Such unwanted systemic effects include cognitive deficits induced by radiotherapy [12], or specific organ toxicity observed through blood measurement of biochemical markers such as liver enzymes and functional factors (e.g., bilirubin and albumin), electrolytes, and blood cell counts (leukopenia) [13]. Nevertheless, assessing the effectiveness of treatment in the short term to avoid risking systemic effects is difficult. The usual endpoints for efficacy are overall survival (OS) and progression-free survival (PFS), but clinicians use surrogate biomarkers for them. Those surrogate biomarkers are mainly related to tumor volume changes or contrast uptake, relying mostly in magnetic resonance imaging (MRI) explorations. The MacDonald set of criteria [14] used for such evaluations is of limited interest, since only the contrast-enhancing area is assessed. However, contrast enhancement after treatment can be due to inflammation, ischemia, or radiation effects. Moreover, the pseudoprogression and pseudoresponse phenomena limit the usefulness of the MacDonald criteria [15,16]. This led the Response Assessment in Neuro-Oncology [17] (RANO) group to work towards a new set of rules adapted to new therapeutic agents (iRANO [18]). Currently, pseudoprogression is still distinguished from true progression through sequential follow-up examinations for at least 3 months, potentially wasting precious time and resources. This situation may become even more challenging for the novel immunotherapy approaches, for which robust non-invasive biomarkers of early response are still missing [19,20]. In summary, conventional MRI alone cannot provide early and confident biomarkers for predicting the response to therapy in GB cases, at least when analyzed in the standard way.

Positron emission tomography (PET)-based molecular imaging has also been used to evaluate the response to therapy in GB cases. However, classical fluorodeoxyglucose (FDG) used in PET is not sufficiently predictive due to various reasons. One major problem is the large background signal of the normal brain, which may complicate the detection of changes within the tumor mass. Additionally, there is a mixture of cell types within tumor masses, mostly microglia/macrophages, myeloid-derived suppressor cells (MDSC), natural killer (NK) cells, and lymphocytes, on top of cancer cells. These cell types can present opposite changes with respect to FDG accumulation in tumors under treatment [21,22], which further complicates matters. Other PET-based biomarkers (immuno-PET) targeting immune system participation have been proposed [23,24,25,26], but none has yet reached routine clinical use for GB.

Accordingly, we clearly need additional approaches—perhaps faster approaches or more informative/specific approaches—for monitoring the response to therapy in GB cases. We wondered whether there would be something changing within the tumor, while its volume is apparently stable, that could inform us about the effectiveness (or lack thereof) of a certain therapeutic agent. As previously pointed out, both human and rodent experimental gliomas are composed of a mix of tumoral and immune system-related cells [27,28]. Among the major contributors to such IS cells’ heterogeneity, volume-wise, tumor-associated macrophages can display either protumor or antitumor functions, and together with MDSCs have the ability to attract T regulatory lymphocytes to the tumor, which may further contribute to the lack of effective immune activation against malignant gliomas. A comprehensive mini-review of this complex matter can be found in [29]. Proper elicitation of the immune system during brain tumor therapy can change the balance of some cell phenotypes [30] or the proportion/infiltration of different immune system elements [31]. The sustained action of the immune system against tumors may lead to local cellular/tissue changes (e.g., cell killing, oedema, appearance of giant cells) that might hopefully be spotted by imaging-related approaches without the need of an invasive biopsy to investigate chosen immune system-related cells through cellular markers. The question is whether this imaging information, whenever available, could be harnessed to improve therapeutic outcomes.

To address the three questions raised in the Introduction, we decided to divert our focus to preclinical animal models of GB, with their advantages and disadvantages. Preclinical models allow for investigating new therapeutic biomarkers with sequential non-invasive follow-up and histopathological validation at chosen timepoints, which would not be ethically feasible with human patients. On the other hand, there are no ideal preclinical GB models. Immunodeficient xenografts are widely used in therapy response investigation [32,33], but the use of such strains prevents the interrogation of the interweaving relationship between adaptive and innate immune system elements and tumor cells. Moreover, xenograft tumor microenvironments usually have a mixture of human and murine cells. This mixture might result in complicated signaling between the host innate IS and foreign tumor cells, hindering proper interpretation, compared with the syngeneic tumor microenvironment [34].

Immunocompetent models such as the GL261 GB (generated from the GL261 cell line, cell code RRID:CVCL_Y003) may be more suitable for this type of study. In this respect, GL261 GB is one of the best characterized immunocompetent preclinical models used in therapy response studies [35,36,37,38,39], as well as in work by our own research group [40,41,42,43]. It is true that there are differences in the mutational load between preclinical GL261 GB (about 4978 somatic mutations in the exome [44]) and human GB (about 14 somatic mutations, on average, in the exome [45]). On the other hand, relapsing human GB may contain a mutational load (between 1000–9000 somatic mutations in the exome) [46] similar to that of to GL261 tumors. In this respect, GL261 perfectly fits the bill for modeling the evaluation of the response when GB tumors relapse and need second-line therapy. GL261 is still a widely used preclinical model for “in vivo therapy studies,” with more than 80 PubMed papers using it in the last 5 years (see recent examples [37,47,48,49]). Moreover, additional effort has been devoted to investigate whether the immunogenic capacity of GL261-originated tumors resembles that of human tumors [50]. From the panel of tumors assessed by those authors (CT2A, GL261, 005 and Mut3), GL261 proved to be a close second to the 005 GB model in terms of resemblance to the immunophenotypic signature of GB patients. In short, these considerations make GL261 one of the valuable preclinical model systems for developing and evaluating imaging biomarkers for the response to therapy in GB cases. A summary of the similarities/differences between GL261 GB and human GB is provided in Supplementary Table S1.

**Figure 1:**
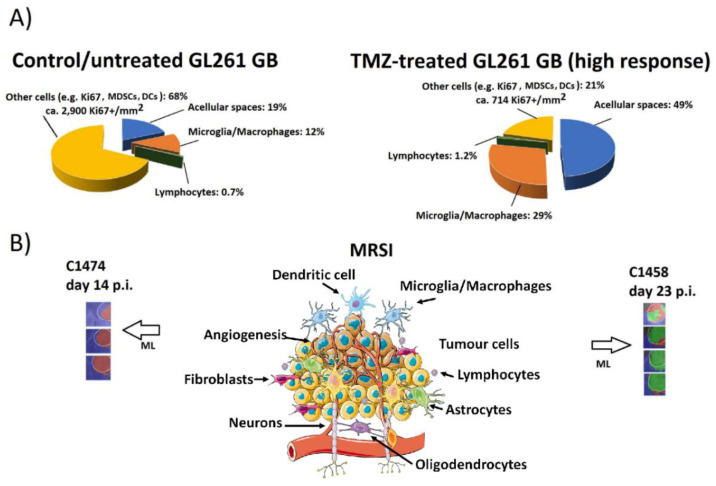
Summary of changes detected in control/untreated and TMZ-treated (high response level, see [43] for definitions) GL261 GB. (**A**) Graph showing average percentages of histopathological features, transformed into volume percentage occupied in the tumor tissue. CD3/Iba-1 immunostaining was used for staining lymphocyte populations and microglia/macrophage populations, respectively. Ki67+ cells refer to GL261 GB tumor cells. The total number of studied histology fields was *n* = 35 to 45 for control and *n* = 22 to 25 for TMZ-treated cases, from a total of four selected mice described in [43] (*n* = 2 control and *n* = 2 TMZ-treated). Response levels were determined through MRSI-based nosological images prior to euthanization. Selected fields corresponded to zones identified as “actively proliferating” (control cases) and “responding” (TMZ-treated cases). (**B**) Scheme of the complex tumor-microenvironment constituted by different elements as described in (**A**), which are variable in control and TMZ-treated, responding cases. At each side, the corresponding MRSI-based nosological images obtained with machine learning approaches (ML) are shown. Left, a representative control case (C1474, 14 days postimplantation (p.i.) of GL261 cells into the striatum of C57BL/6j mice). Right, a representative TMZ-treated, transiently responding case (C1458, 23 days p.i.) from [56]. Please note that each studied mouse was identified in the cited studies with a unique code (CXXXX) where XXXX was a correlative number. The color code in the nosological images was red for proliferating tumors, green for responding tumors, and blue for normal tissue. The Tumor Responding Index (TRI, please refer to Supplementary Material section) was calculated and achieved values of 0% (control case) and 81.8% (TMZ-treated, responding case), most probably reflecting a combination of the cellular/tissular elements changes shown in (**A**).

In this respect, we have reported that using GL261 GB growing in immunocompetent C57BL/6j mice resulted in metabolome pattern changes of tumors under therapy that correlated with survival, during time periods of days-weeks, in which no tumor volume changes were visible (<10%) [43]. Those metabolome pattern changes were spotted with magnetic resonance spectroscopic imaging (MRSI), which was sampled with a frequency of ca. 2 days. With this dataset, we were able to measure the response level in terms of the Tumor Responding Index (TRI). This objective measurement was achieved through applying machine learning approaches over all MRSI data vectors and categorizing tissue as “responding,” “control/actively proliferating,” or “normal/unaffected,” codified by different colors (Figure 1B and Supplementary Materials Figure S1). The main metabolites involved in these spectroscopic pattern changes associated with the GL261 GB response to therapy have been described by us in [51,52] and are also summarized in Supplementary Materials Table S2. The signals with the greatest contribution to the discrimination were related to mobile lipids, lactate, and polyunsaturated fatty acids (PUFAs), as well as myo-inositol/glycine. Metabolites such as PUFAs were described in situations of cell apoptosis (e.g., [53]), while metabolites such as lactate could be associated with both tumor-associated macrophages metabolism [54] or tumor cell metabolism (e.g., in case polyploid cells arise [55]). However, there are more subtle changes expected in these situations, which would not be straightforward to measure and analyze separately. In this sense, the investigation of whole pattern of changes may increase robustness (whole vector instead of single metabolites) and decrease subjectivity (user-related differences in processing-quantification, metabolite selection, and normalization differences, among others).

**Figure 2:**
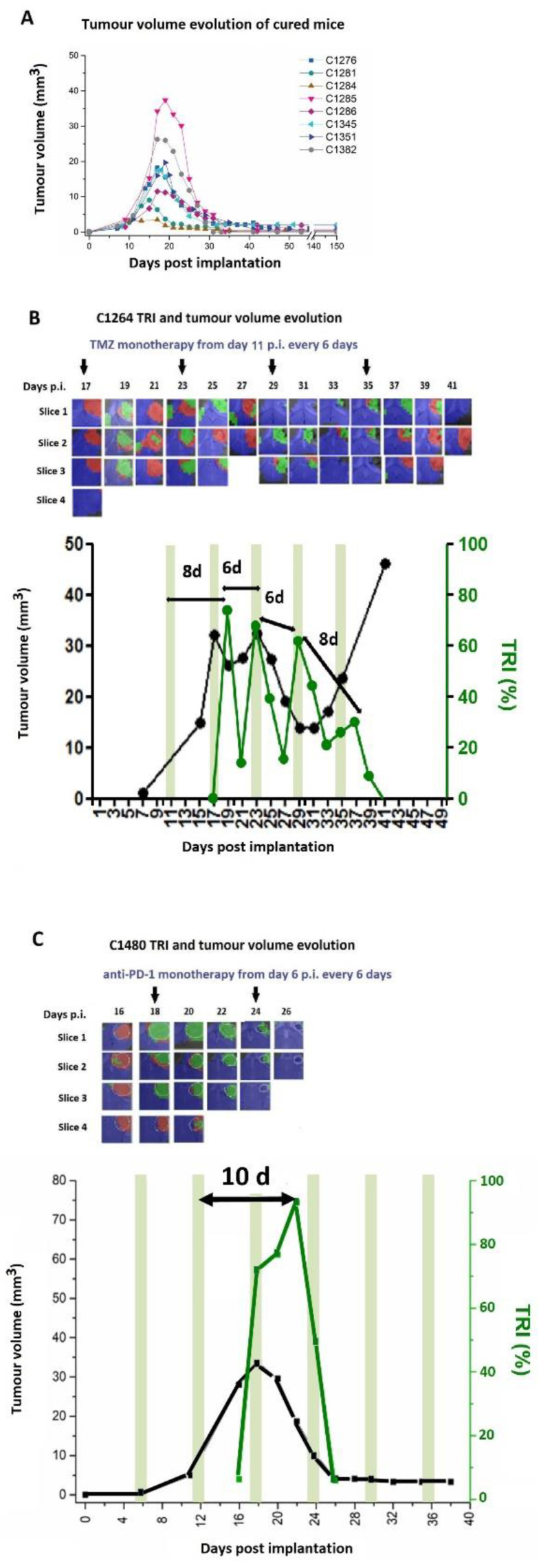
(**A**) Tumor volume evolution of GL261 tumor-bearing mice declared cured from an Immune-Enhancing Metronomic Schedule (IMS)-TMZ treated group (*n* = 8), described by us in [57], consisting of an every-6-day administration. Joint graphical representation of MRSI-based nosological images and tumor volume evolution for (**B**) case C1264, treated with IMS-TMZ and (**C**) case C1480, treated with anti-PD-1 immunotherapy in IMS protocol. Tumor volume in mm^3^ (black line, left axis) and the percentage of responding pixels (TRI, green line, right axis). See Section 2.1 and the Supplementary Materials for a description of the TRI calculation. Green-shaded columns indicate therapy administration days (TMZ and anti-PD-1 immunotherapy respectively). TRI cycle duration (i.e., therapy administration to next peak maxima) is highlighted in every image. In the upper part of the images, the evolution of the nosological images is shown superimposed onto the T2w-MRI for each slice for chosen timepoints. Vertical black arrows indicate days of therapy administration. (PD-1: Programmed cell death protein 1). Please refer to Figure 1′s caption for an explanation of mice code alphanumeric identification. Figure reproduced with permission from [57].

The TRI was then calculated taking into account the percentage of pixels classified as “responding” (green) divided by the total pixels counted within the tumor mass. The TRI values displayed an oscillatory behavior with ca. 6-day period in mice [41,57] (Figure 2B,C and Supplementary Materials Figure S2). Such oscillations have not yet been detected in GB patients going through MRSI, MRI, or PET acquisition, for reasons that will be discussed further on.

Thus, a new time frame window, days/weeks, to monitor IS-associated effects in GB therapy may have been opened. Then, we wondered what was the origin of the oscillatory pattern giving rise to the oscillating TRI, and what could we use it for? Pattern changes upon treatment in the case of the GL261 GB were shown to arise in local changes induced by therapy (Figure 1A). Previously, we evaluated the effect of chemotherapy (TMZ and cyclophosphamide (CPA)) [40,57]) and immunotherapy with immune checkpoint inhibitors ((CKI) anti-PD1, [41]), either as monotherapies or in combination. However, as stated above in the Introduction, all major therapies against GB seem to involve recruiting the IS to strike tumors. Thus, it would be sensible to speculate that (transiently) efficient therapies will raise comparable cellular and metabolomic changes in responding GB tissue.

**Figure 3:**
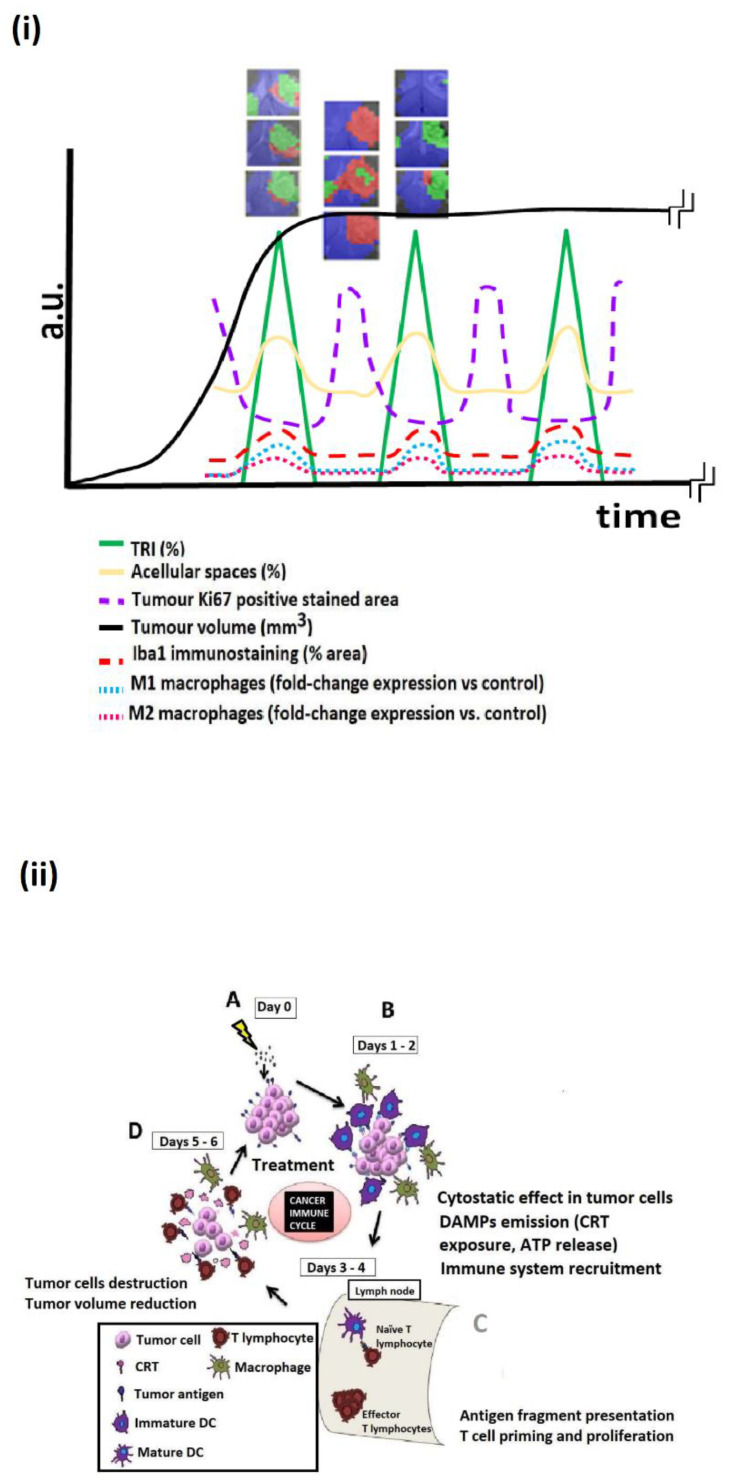
(**i**) Tentative summary of the main cellular/molecular features changing during GB treatment and the associated changes in the MRSI-detected metabolome pattern described by our group. Representative nosological images were obtained from Immune-Enhancing Metronomic Schedule (IMS)-TMZ treated case C1264 [57] during a transient response to therapy (i.e., stable tumor volume, black solid line). The TRI oscillations (green solid line) are an average representation of transiently responding cases reported in our previous work [43,57]. Acellular spaces (dark blue solid line), the Ki67-positive stained area (purple dashed line), and the Iba-1 stained area (brown dashed line) were estimated from fields of histopathological slides from high-level response (*n* = 27 fields) and control cases (*n* = 40 fields) described in [43]. M1 and M2 macrophages’ gene level expression changes (light blue and pink dashed lines) were calculated from NOS2 and CD206 obtained from IMS-TMZ treated and control tumor samples (*n* = 10 each) described by us in [56]. (**ii**) Schema of cancer immune cycle (reproduced with permission from [57]) starting from (A) treatment (e.g., in this example, IMS-TMZ), which triggers immunogenic cell death/damage, (B) release of DAMPs (CRT, calreticulin) [72] and antigen presentation to host immune system cells, followed by (C) T-cell priming and proliferation, which (D) infiltrates the tumor site and kills tumor cells.

In short, what are the cellular changes taking place in treated GL261 GB upon response, as compared to control untreated tumors? Figure 1 summarizes those detected changes. Our results [43,56] show that in a fast-growing GL261 GB tumor before treatment, about 68% of the tissue volume is occupied by fast proliferating tumor cells (average 2900 Ki67+ cells/mm^2^) and other non-tumor, non Iba1+ cell types, such as myeloid-derived suppressor cells (MDSC) [28]. The rest of the volume is mostly represented by innate Iba1+ IS cells (microglia/macrophages, 12%), lymphocytes (below 1%), and acellular spaces (also dubbed by other authors microcystic volume [58]) (19%). On the other hand, in a treated/responding tumor, the extreme opposite pattern consists of only 21% tumor cells (plus MDSC), with decreased proliferation (average 714 Ki67+ cells/mm^2^). Moreover, although a similar lymphocyte volume-occupied percentage as in controls is observed (above 1%), there is a clear increase in the innate Iba1+ IS cells (29%) and acellular spaces (49%) content. Proliferating and growth-arrested cancer cells are known to display metabolome differences, especially under therapy [59,60,61]. In addition, TMZ treatment is known to trigger the appearance of giant tumor polyploid cells described as quiescent and displaying an increased glycolytic metabolism, releasing more lactate than diploid tumor cells [55,62]. Microglia/macrophages under different polarization statuses also have different metabolomic patterns [54,63]. In this respect, changes in the microglia/macrophage polarization (M1 vs. M2) have been described in both GB [64,65,66,67] and, more precisely, in GL261 GB [56,68,69,70]. Then, the composition of the cell types, their status and tissue architecture, and the changes upon treatment can together contribute to metabolome pattern changes that lead to measurable changes in the TRI (Figure 1B). Furthermore, as mentioned above, changes appear cyclically over time, in ca. 6-day periods, as shown in Figure 2 and Figure 3i and Supplementary Materials Figure S2. The role/relevance of less-investigated types of macrophages, such as SiglecF+ [50,71], should still be clarified.

Accordingly, the changes observed in the cellular characteristics of the tumor mass within a time frame of days may reflect the therapy effects over tumor cells and also the action of the recruited IS on the tumor. On the other hand, the immunogenic cell damage (see below) can induce changes in the IS-related populations, which can bear different metabolomic fingerprints [54]. These are detectable by the metabolome pattern registered in MRSI acquisitions, and transformed into response (nosological [73]) images by machine learning (ML) postprocessing [52,74]. See also the Supplementary Materials Figure S1. This pattern of behavior correlates with the response, survival, and cure of some mice, and even with the immune memory in the cured animals upon re-challenge [40,41,43,57]. In this sense, it is also worth remembering that the overall cancer immune cycle lasts around 6 days [75,76] (Figure 3ii), corresponding to a circaseptan rhythm also described in [77] that matches the oscillations detected in the metabolomics pattern. Continuous administration of anti-proliferative therapy over days of T-cell amplification in the proximal ganglia (Figure 3ii(C)) could impair proper IS action against the tumors and should be avoided.

Untreated animals may transiently show tumor regions with MRSI patterns compatible with IS attack. Additionally, we have observed that GB tumors escaping from therapy may also show such regions [43,57]. What they do not seem to show is oscillatory periodic behavior, such as responding tumors display. We have proposed [40,41,43,57] that this oscillatory behavior of the MRSI-based response biomarker is caused by the cancer-immunity cycle [76], in which therapy at day 0 increases DAMPs release by the tumor cells (Figure 3). This will attract naïve dendritic cells (DCs), which will then travel to proximal ganglia, activating naïve lymphocytes. These lymphocytes will proliferate and migrate back to tumor tissues, to act against cancer cells displaying antigens initially sampled by DCs (within 5–6 days of therapy administration). Microglia/macrophages, also attracted by DAMPs, will be polarized to the M1 subtype to collaborate with cytotoxic T lymphocytes (CTILs). Once the CTILs become exhausted, tumor cells will regain proliferation, until the next wave of therapy and IS recruitment. This metastable situation will lead to some tumors being wiped out by the IS after one or several cycles of therapy (Figure 2A), while other tumors will escape from therapy (Figure 2B). This escape may be due, for example, to an unchecked increased PD-L1 expression [57,78], unless anti-PD1/PD-L1 CKI is used (Figure 2C and [79]). Changes in the MDSC cell subtypes during therapy [50] may also contribute to this MRSI-detected oscillating pattern.

Accordingly, a predictor based on the detection of an oscillatory pattern of response by MRSI from preclinical models should provide an early hint, within 1–2 weeks, that the therapy being used is effective and should be maintained. On the other hand, the absence of such oscillatory behavior should definitely warn researchers that the tumor cells are (or eventually become) resistant to the therapy being applied. When this therapy is translated to a clinical setting, it should allow for an evidence-based decision to discontinue therapy and move as soon as possible to a second-line therapeutic strategy, which could be further evaluated for performance by the translated imaging-based predictor of response in 1–2 weeks.

### 2.2. Why Are Such Biomarkers of Efficacy Not Yet Available?

**Figure 4:**
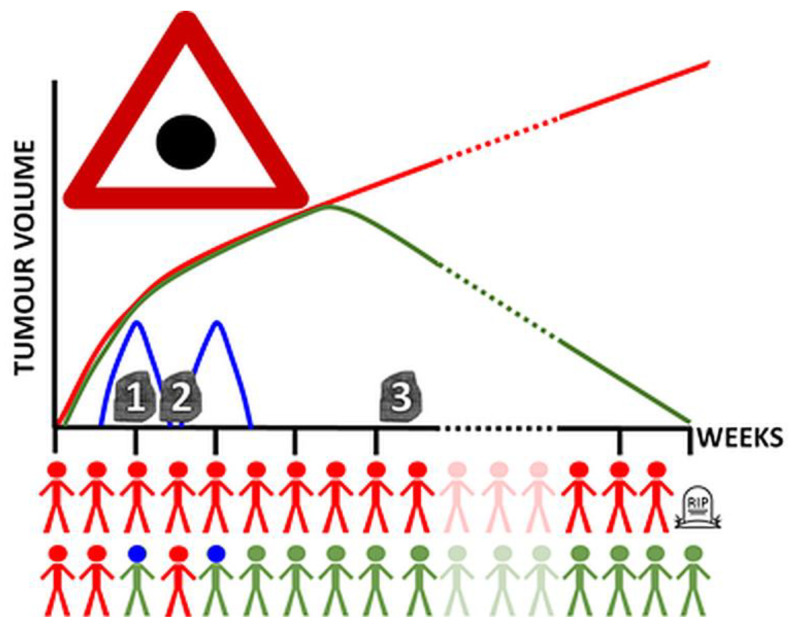
Our hypothesis in a graph. Patients that do not respond to therapy (red) will show a sustained increase in tumor volume with time. Patients that respond to therapy (green) will show a decrease in tumor volume at some timepoints, but before that, the immune system (blue curves) will have successfully attacked the tumor (indicated by the person with a blue head when the immune system activation is maximal). Note the period of one week of the blue curve. If we only measure tumor volume, we would be in a temporal blind spot (the red triangle with the black dot). Therefore, we should sample our biomarker at the precise time (1), since the wrong times (points 2 and 3) would be either uninformative or too late.

There are several reasons that could explain the lack of a widely accepted early biomarker of response to therapy in GB cases (first for preclinical models and later for patients) [16]. The proposal of this article emphasizes the relevance of one of them—the oscillatory behavior of the changes, in the time scale of days, observed in the metabolome and tissue structure of GB under treatment. This caveat—the absence of an earlier biomarker and possible artefacts due to sparse sampling of patients’ condition—is schematized in Figure 4.

As far as we know, there are no human GB studies sampling tumor data with suitable frequency for observing such oscillatory behavior, and whether these data could be used to evaluate response efficacy. The frequency of patient data acquisition is usually between 1.5–3 months, both for MR- or PET-based explorations [80,81,82,83], but single timepoint explorations are also reported [25]. These sampling protocols do not allow the proper extraction of meaningful variables about IS efficacy when a possible oscillatory pattern (Figure 3 and Figure 4 and Supplementary Material Figure S2) for changes taking place locally in the tumor may arise, within a time frame of a few days (2–3 days may represent a relevant difference). A single study reported evaluation of GB patients with MRI and peripheral blood markers (i.e., SDF-1α levels) on a weekly basis during the concomitant radio/chemotherapy part of the Stupp protocol, 6 weeks [84]. These authors found associations between increased marker levels during late radiation (week 5/6) and worse outcomes, which is in line with the SDF-1α role in glioma cell survival [85]. Moreover, a recent immuno-PET preclinical study on subcutaneous MC38 adenocarcinoma evaluated the content of cytotoxic infiltrating lymphocytes (CTIL) and global myeloid cells (MDSCs) in the tumor with a 4-day sampling period after anti-PD1 therapy for up 27 days [26], but did not interpret the changes in the parameters evaluated considering possible oscillations, e.g., CD11b+ cells (myeloid, MDSC, subpopulations marker) may display a clear oscillation every 8 days, which was not considered by the authors. In this respect, our Figure 4 hypothesis would have allowed the authors in [26] to look at their results from a different perspective.

The lack of high frequency sampling data is evidently due to ethics restrictions related to repeated explorations in patients when added value is unclear [86]. Other possible reasons may be the associated costs of repeated explorations, difficulties related to the tracer used (PET), or difficulties related to adequate data postprocessing. Still, added value for high frequency imaging explorations during a restricted period of time, just to gauge therapeutic efficacy, needs to be first shown preclinically to justify embarking on translational protocols with patients [16].

A different option to facilitate high-frequency tracking would be targeting periphery evaluation of possible IS biomarkers of response that may approach an answer to the therapy-efficacy question. In this respect, Rühle et al. [87] reported distinct immune cell subset content modulations in the blood of a single GB patient over 17 months of follow-up (blood sampling every 4 weeks and MRI evaluation every 4–17 weeks). Weekly changes in the immune cells’ subsets were detected by the authors. In this respect, the decreased circulating CD4+/CD8+ ratio was clear at weeks 14–18 after the start of the Stupp protocol [1,2], when the patient was already receiving TMZ monotherapy alone. This CD4+/CD8+ ratio decrease preceded the tumor mass’s transient disappearance at week 18, in which a concomitant CD4+/CD8+ ratio peak high was detected. This increase in the ratio was observed far before relapse at week 29. Additionally, Alban et al. [88] described significant CD8+ circulating T cells’ increases at 2 and 8 weeks after surgery, followed by standard radiotherapy and chemotherapy, in a longitudinal study of the blood of six GB patients. Unfortunately, none of those studies evaluated short-term changes (days) in the blood of patients that may correlate with the transient response to therapy in GB cases. On the other hand, more than a decade ago, Coventry et al. [89] examined patients with different cancer types and described variations in the C-reactive protein (CRP), an acute-phase plasma protein that can be used as a marker for activation of the immune system, which may hint at treatment success. In their study, CRP measurements were shown to present cyclical oscillations with a period of approximately 6–7 days. The authors proposed CRP as a surrogate therapeutic biomarker that was described as being related to tumor T-effector and T-regulatory clonal expansion and activity. Unfortunately, the broad role of the pathological situations that can relate to CRP fluctuations constitute a possible confounding problem for the CRP measurements’ use in this regard [90]. Recent work along these lines [91] also suggests that a mean of 7.3 days (range 6.5–10 days) in the oscillation of immune cycle blood biomarkers (High Sensitive C-Reactive Protein, Lactate Dehydrogenase, and Lymphocyte/Monocyte Ratio) could be used to synchronize partial radiotherapy of bulky body tumors with the immune cycle’s homeostatic oscillation. Finally, Kitano et al. [92] recently described the study of micro-RNA (miRNA) in urine samples from brain tumor patients with the potential to investigate malignant transformation. In this study, they focused on pre-surgery samples, without investigating possible fluctuations during therapy. However, the fact that those miRNA could also reflect the influence of the environment and immune system cells involved with the tumor points to this approach being worth investigating and constituting an appealing option. In another study regarding urine samples, Tandle et al. [93] investigated changes in the metabolomic profile after radiation treatment in GB patients, but its correlation with patient outcomes was not explored in their work.

Ideally, local evaluation of changes within the GB tissue should be a more precise biomarker than periphery evaluations of immune system status [28,94,95]. For such local evaluation, non-invasive methods are ideally suited, such as MRI (also MRSI) and PET. Why has this approach not yet been successfully applied to patients? Several explanations can help in understanding this fact. In the first place, as stated above, why should high frequency sampling be considered when no benefit for such sampling has yet been proposed for patient prognosis? [86]. Second, even if this was considered, acquiring MRI, MRSI or PET scans every 2 days for a long period of time has never been attempted because of ethical issues, costs, and/or possible related iatrogenicity.

To address the first problem, a reasonable and sensible initial approach seems to be acquiring such types of data from preclinical immunocompetent models. Once the beneficial effect of a non-invasive biomarker is demonstrated, and the minimum time length required for robust prediction of therapy response is assessed, a clinical proof-of-concept study could be launched. Are such longitudinal studies with preclinical models available? Only in a limited fashion. Most studies monitoring GB treatment with high frequency, i.e., 1 day in Lazovic et al. [94], restrict this approach to short follow-up periods (4 days’ total in Lazovic et al. [94]). In turn, this type of short follow-up prevents evaluation of the effects on survival or on the oscillatory behavior of the chosen biomarker (MRI in their case) in a time range of weeks. Most studies investigated few timepoints; for example, two timepoints 1 week apart in Rygh et al. [95] did not allow for evaluation of possible oscillatory behavior. 

There are two additional items to consider in order to understand the challenges related to preclinical studies when it comes to mimicking IS attack on GB for evaluating imaging biomarkers. First, many previous studies used xenograft models, sometimes in non-orthotopic environment, i.e., subcutaneous models, mostly in mice [96,97,98]. Depending on the case, lymphocytes may be absent from the system or the immune microenvironment may be largely different from the brain. For example, the adaptive immune system is lacking in varying degrees in immune-deprived mice models and, accordingly, no cancer-immune cycle is expected to be comparable with immunocompetent mice, or humans. In other words, the oscillatory behavior detected in treated GL261 GB growing in immunocompetent C57BL/6j mice may not be detectable while evaluating human GB cells growing in immune-compromised mice brains. Accordingly, it is worth remembering that studies in immunocompetent models should be performed to better mimic human conditions. A second relevant item to consider is that any therapy with cell cycle-arresting properties, such as radio- or chemotherapy, when administered daily, will damage lymphocyte amplification at the proximal ganglia, compromising immune cycle completion [99,100,101]. A way out of this problem for preclinical work is synchronizing therapy with immune-cycle length (6–7 days), as in the immunoenhancing metronomic strategy [40,41,57] (e.g., Figure 2). Once the biomarker and the derived predictor are shown to be robust in preclinical models of GB, we will be faced with the second problem—high frequency sampling and its limitations in humans—which is considered in Section 2.3.

### 2.3. How Can We Produce the Needed Biomarkers?

First, as mentioned in Section 2.1, it should be demonstrated, preferably in preclinical immunocompetent models, whether major therapeutic strategies for GB are able to significantly increase survival in those models (or to cure them) and to produce metabolome/cellular/structural changes compatible with IS attack on tumors. Examples of therapies to be studied are radiotherapy, chemotherapy, surgery, targeted therapies, immunotherapy, and antiangiogenic agents. Moreover, in cases of cured animals, immune memory against further exposure to the same tumor type would also be expected, but it should be confirmed experimentally. Aside from the well-known GL261, other immunocompetent models should be tracked for robustness; for example, SB28, with lower mutational burden [44], or the CT2A, which is considered less immunogenic than GL261 [50].

Thus, if the immune system is simply “called for duty” by each one of the (transiently) successful therapeutic strategies, predictors developed from one cohort of mice treated with a given therapeutic agent should be transposable to predict the efficacy of other therapeutic agents, since tumor cells and infiltrating immune system cells would be the same (Figure 1).

Provided an adequate set of preclinical models and suitable therapies are available, the three major non-invasive imaging methods of therapy response—MRI, MRSI, and PET should be applied at short intervals (days) to individuals. Additionally, possible complementary peripheral biomarkers in blood [102] (immune cell profile [87,88], cytokine/chemokine content [103], and plasma cell-free DNA [104]) can also be accrued. Still, an appropriate translational perspective must be exercised. Which set of measurements should be best for future acquisition from patients in order to produce relevant information about responses? Or, in other words, how many sampling timepoints are needed to predict the evolution of the tumor in the short term? This is still an open question that needs an initial preclinical answer. Figure 2 and Figure 3 suggest that 6–7 locally informative timepoints from a mouse, after therapy administration and over a 2-week period, should be enough. Specifically, we need to investigate the minimal number of explorations needed to detect oscillatory behavior robustly, numerically, and by a predeveloped predictor.

Next, which imaging method would be better suited for the required purpose and also for clinical translation? Again, the methods must be tested, and challenges related to technical obstacles and infrastructure need to be considered for translation [16]. If we accept the information as shown in Figure 1, we would expect cyclical changes in various parameters—notably, percentages, proliferation, and polyploidy, of tumor cells; percentages and polarization stages of macrophages and MDSCs; and changes in the extracellular compartment volume. All of these parameters are amenable to detection by the three major imaging methods mentioned before. PET is much more sensitive than MR, and accordingly, it may interrogate changes in low-percentage cell type populations (e.g., activated T cells) that are not directly observed with MR. Still, if several different questions need to be asked, for example about the proliferation of tumor cells, the activation state of lymphocytes, or the polarization stage of macrophages, different tracer injections may be needed for PET imaging, thereby increasing the required number of explorations. MR is less selective about what is being observed, but on the other hand, it may allow observing the joint effect of relevant response-associated information in a single exploration [16]. This will be fine, provided that the information accrued is sufficient to produce the required translational answer. Most probably, MR and PET explorations will complement each other (see also Chawla et al., 2021 [20]). It seems sensible to expect multi-timepoint explorations in days to be MR-based, while confirmatory single timepoint explorations, asking single questions, should be based on PET scans.

Let us focus on which questions can be addressed by the MR-based methods, MRI and MRSI. As reviewed in Section 2.1 and Section 2.2, preclinical MRSI-based protocols allow us to monitor the short-term oscillatory behavior of the GB metabolome, which agrees with IS productive recruitment in the tumor. Moreover, depending on the therapeutic protocol used (IMS-TMZ or anti-PD-1 [40,41,57]), we can also observe the cure of the studied mice, even with immune memory. This has required MRSI explorations every two days during the monitoring period of interest. Could similar information be obtained from MRI alone? The radiomics approach [105,106], which uses different types of MR images on a pixel-by-pixel basis, hints that this could indeed be possible. Radiomics proved capable of extracting imaging features to differentiate pseudoprogression from true progression in a retrospective study of treated GB patients explored by MRI [107]. However, information on the possible oscillatory behavior of the relevant features was not considered in that work. If features used in [107] were robust enough to predict responses to different therapies, a single timepoint analysis should be sufficient for a therapy response assessment. This approach comes with a caveat, however. Clinicians must wait until imaging conditions compatible with pseudoprogression appear in order to apply the developed radiomics-derived predictor. Ideally, we should require a single (or a few) timepoint predictor that works properly, regardless of the oscillatory influence of the immune cycle—or robust enough in spite of it. We recently reported a step towards this approach, for preclinical GL261 GB retrospective data analysis using MRI radiomics, compared with source analysis of MRSI data [108]. Additionally, quantitative information has been derived from contrast-enhanced MRI data about glioblastoma tissue heterogeneity, the “habitats” [109] that may bear relevance to therapy response assessment [106,109], provided the oscillatory behavior described in this manuscript is taken into account.

The study of brain tumor metabolism has been also investigated through hyperpolarized MR. This is particularly interesting, since in MRS(I) only metabolites with concentration of around 1 mM and beyond can be detected. Hyperpolarized MR provides signal enhancement of several orders of magnitude and bears the potential to interrogate in vivo metabolic fluxes through pathways such as glycolysis and the Krebs cycle. Since relevant differences are expected in untreated tumors vs. tumors responding to therapy, this approach has been explored, especially in preclinical settings [110,111]. In this sense, while evaluating responses to chemotherapy, the pyruvate-to-lactate conversion changed before any reduction was described as taking place in tumor volumes in brain tumor-bearing xenografts [111]. Radiotherapy treatment was evaluated with hyperpolarized MR in [110], with sampling every 7 days after treating tumor-bearing mice (xenografts with human-derived glioma sphere-forming cells) at days 25 and 27 p.i. In this scenario, pyruvate-to-lactate conversion presented an increase at day 34 (7 days after the last radiotherapy session), but the chosen sampling frequency does not allow us to infer any possible oscillations that could be taking place. It is also worth mentioning that although the GE Healthcare’s polarizer has been approved for the clinical use of hyperpolarization and has been used in phase I trials [112], this approach requires access to complex equipment that is not available in most clinical centers, and further complementary studies may be needed to investigate added values and to carry out a cost-benefit assessment.

How could PET explorations help in complementing this type of MRI-based predictor? Let us assume that MRI-based predictors would indicate timepoints, in which relative increases of CD8+ lymphocytes, total macrophages, and M1-polarized macrophages are expected under successful therapy (Figure 1 and Figure 3). Still, the MRI/MRSI data would not quantify individual changes in those cell types, although some inroads into this have been proposed for macrophages [113]. On the other hand, all three cellular parameters can be relatively quantitated non-invasively by PET [23,24,25,26,114]. Thus, PET could confirm GB patients’ response status proposed by MRI/MRSI (see also [20]), allowing evidence-based decisions by the clinical review team as to whether the patient should stay in the initial therapy or discontinue it and switch to a second line, much faster in comparison with currently established follow-up protocols.

### 2.4. Limitations of the Present Hypothesis Proposal

The present proposal is mostly based on results obtained with a single preclinical immunocompetent model (GL261), and with a limited set of therapeutic protocols. Thus, only chemotherapy (TMZ, CPA), targeted therapy (CK2 inhibitors), and immunotherapy (anti-PD1) have been used by the authors. It would be relevant to test whether the oscillatory behavior of the MRSI-detected pattern still holds with other major therapeutic strategies used in patients, e.g., surgery and radiotherapy, that are used to treat mice. Additionally, extension of this work to less immunogenic mice models (CT-2A, SB28) would allow us to gauge the robustness of the GL261 described observations.

An additional word of caution may be raised, since the GL261 preclinical model does not fully emulate the whole human mutation panel, and this could impact on the observed MRSI spectral pattern. An example is the mutations in the isocitrate dehydrogenase 1 (IDH1) gene, which are more common in secondary GB [115]. This essentially leads to differences in the basal (untreated) pattern regarding the 2HG metabolite [116], which can exhibit significant changes depending on the therapeutic approach being used. Since in this work we have not explored any preclinical model bearing the aforementioned mutation, it is still to be determined which model would indicate the specific impact of a given metabolomic change over the whole pattern, provided the rest of the changes were consistent. Still, mutations such as ATRX loss [117] are not observed in parental GL261 cells but are indeed considered as drivers to higher grade gliomas [118] and can have a significant impact on the immune responses after therapeutic approaches (and hence, on the expected MR-based biomarkers). It was beyond the scope of this work to explore the large panoply of particular situations that may finally derive into differences in the registered MRSI-based pattern under treatment. We wanted, rather, to hypothesize that regardless of that consideration, there may be a temporal, oscillatory pattern change, a factor that is currently being ignored.

Moreover, it was not our intention to provide within this text a comprehensive review of other competing methodologies for providing non-invasive imaging biomarkers of response to therapy in GB cases, but only to provide relevant examples of those approaches that have been highlighted. The interested reader is advised to check other articles/revisions in this regard [16,18,19,20,21,22,80,81,82,86].

Limitations of the MRSI in its clinical use. Currently, MRSI is not being routinely used in the clinical pipeline, either for diagnosis or for follow-up strategies, except in very specific circumstances. A survey performed in 2018 [119] regarding glioma imaging and including 220 centers (oncological centers, general hospitals, and academic hospitals) summarized that 80.4% of the institutions used MRS in clinical brain tumor imaging, but rarely as part of their routine protocols. MRS acquisition was mostly undertaken when based upon a request or for a specific indication, when related to the distinction of tumor from non-neoplastic conditions, or for tumor grading. Differentiating therapy effects from tumor recurrence was a less common use.

This essentially means that there are many clinical centers around the globe with the potential to perform MRS(I), but which are not currently exploiting this potential option in daily practice. Several reasons can help to explain this fact. First, as opposed to MRI, MRS(I) does not yet have a standardized file format, differing among vendors in regard to the final outputs. Hence, sharing and exchanging files is not straightforward, as it is for MRI. Moreover, validation of approaches across different centers can be challenging, due to variations in acquisition and analysis methods [16]. Second, the spectroscopic information is initially based on spectra with different chemical shifts, requiring a strong biochemical/physics background for use. While radiologists are accustomed to interpreting anatomical features, this may be harder in cases of spectral pattern-based information. Third, for the approaches mostly used in the literature, based on the quantitation of main metabolites (or ratios), mastering of different processing/postprocessing/quantitation software may be required (e.g., jMRUI [120,121]) or Tarquin [http://tarquin.sourceforge.net/, last accessed 10 February 2022). Moreover, MRSI acquisition is more challenging than MRI acquisition. Specifically, suitable homogeneity is required over large volumes in order to achieve sufficient spectral resolution and good water suppression. Further, scalp lipid suppression is needed due to the vicinity of the skull region. The fact that the observed metabolite signals have lower concentration (and, hence, lower SNR) compared with water also poses challenges in spectra acquisition and interpretation. A methodological consensus was revised and discussed by Wilson et al. in [122].

Last but not least, strong evidence for the added value of spectroscopic information over MRI is still lacking, being restricted to few studies (e.g., [123,124]).

The acquisition of MRSI is indeed approached in some clinical centers, but most of them are academic hospitals involved in research with protocols that are not fully integrated into standard clinical practice pipelines and, in general, use quantification (or the generation of a metabolic image) of few metabolites or ratios, such as NAA, Cr, Cho, glx, m-Ino/gly, GABA, or 2HG [122,125,126,127,128,129,130,131,132,133,134,135]. Recent reviews suggest that 3D-1H MRSI sequences are useful in evaluating therapy response, since they are able to map metabolomic information from peritumoral regions [20], but these types of sequences are challenging and are not widely available in regular clinical centers.

Regarding GB/glioma therapy response follow-up or relapse, again most centers have applied these protocols for research purposes, and centered on few metabolites or ratios (e.g., Cho, NAA, and phospholipid metabolites) [136,137,138,139,140], while using the whole pattern is seen in few studies (see, e.g., [141,142]). Metabolome pattern changes during therapy response can be subtle and can happen in several metabolites at once and in different directions, increasing or decreasing. This pattern will be not easily spotted in studies limited to few metabolites or ratios. Thus, robust and standardized whole pattern studies may be needed in this sense, especially because in the case of metabolomic changes related to immune system activity, more than one MRSI exploration may be required, with the timing adjusted to the expected immune system local changes (Figure 4).

Moreover, even in case of robust studies based on pattern recognition approaches with suitable added value information, in order to be used by clinicians in daily practice, they may need to be integrated in a user-friendly system, offering fast, reliable, and easy-to-interpret outputs. In this sense, we are confident that outputs such as nosological images [73,143] can be of great help and, provided their added value is confirmed in hospitals, would be appreciated and incorporated by clinicians.

## 3. Conclusions

Following the questions formulated in the Introduction, the following conclusions are indicated. (1) We need imaging biomarkers for assessing the efficacy of IS participation in GB therapy responses. Such assessments may be the earliest hint of (transient) therapeutic success (or lack thereof), but they are mostly ignored in the current follow-up pipelines for patients. The current pipelines, even when “adapted” (as is the case with iRANO) still represent a waste of precious time for patients who have a pathology with a dismal prognosis. Reducing this time gap should help them. (2) However, needed IBs are not yet available, because most follow-up approaches have been based on MR imaging criteria (MacDonald, RANO, or RECIST) or other approaches such as PET, but the required sampling frequency to detect circaseptan rhythms (which our preclinical results demonstrate) was not used. (3) Finally, these MR/PET biomarkers must be refined through systematic preclinical work in order to fully understand the cellular/molecular events behind them and the minimum amount of data timepoint sampling needed for the robust prediction of successful IS anti-tumor attack. Additionally, the optimization of cross-confirmation between MR and PET acquisition may be advantageous.

Considering the preclinical and clinical evidence that has been provided, it becomes clear that the clinical pipelines for GB therapy follow-up may be optimized. Single shot follow-up non-invasive acquisitions may miss information or, even worse, provide misleading information if oscillations related to IS are not taken into account. Adjusting follow-up protocols would be of great help to patients, since non-effective therapies can be discontinued early and other approaches may be attempted, with increasing possibilities of enlargement of PFS, and to health systems, saving time and resources instead of maintaining ineffective therapeutic strategies with few or no effects on patient survival or quality of life. This proposal is not confined to GB treatment with “classic” immunotherapeutic approaches, since it is widely accepted that most of the GB efficient therapies are able to elicit a host immune response. Neglecting this effect and its possible impact on imaging and spectroscopic features may lead us to underestimate the advantages of these therapeutic approaches with respect to GB, and definitely will not help improve patients’ outcomes.

## Data Availability

Data from the manuscript’s authors are accessible upon reasonable request to the corresponding author. Other data from the cited literature must be accessed through the corresponding authors of such work. The data are not publicly available due to raw uncurated datasets including MRI and MRSI of dozens of mice are indeed too large and complex for unsupervised public sharing. Prior consultation between interested groups and data originators is needed for sensible use of those data.

## Supplementary Materials

The following supporting information can be downloaded at: https://www.mdpi.com/article/10.3390/metabo12030243/s1, Figure S1: Summary of steps performed for the nosological images calculation mentioned in this work; Figure S2: Hypothetical scheme for the rationale behind changes in the nosological images coding for response in MRSI of IMS-TMZ treated GB GL261 bearing mice; Table S1: Main similarities/differences between GL261 tumors and human GB tumors; Table S2: Metabolites (ppm and tentative assignment) associated with the main changes observed in the metabolomics profile of control and TMZ-treated GL261 GB. References [144,145,146,147,148,149,150,151,152,153,154,155,156,157,158,159,160,161,162,163,164,165,166,167,168,169] are cited in Supplementary Materials.
