# Supplementary material for: Establishing Imaging Biomarkers of Host Immune System Efficacy during Glioblastoma Therapy Response: Challenges, Obstacles and Future Perspectives
Source: Metabolites. 2022 Mar 14;12(3):243. doi: 10.3390/metabo12030243 (PMC8950145; doi:10.3390/metabo12030243)
Supplement: Supplementary file 1 [file metabolites-12-00243-s001.zip › metabolites-1608473-supplementary.pdf]

metabolites

MDPI

Hypothesis

Supplementary Material

Establishing Imaging Biomarkers of Host Immune System Efficacy during Glioblastoma Therapy Response: Challenges, Obstacles and Future Perspectives

Ana Paula Candiota and Carles Arús

Supplementary Introduction

MRSI analysis: outline of NMF methods

Non-negative matrix factorization (NMF) methods are multivariate data analyses designed for estimation of meaningful components (also known as sources), which originate from non-negative data. Standard NMF methods decompose data "X" into 2 non-negative matrices: sources ("S") and mixing matrices ("A"). The divergence between X and S*A is measured by cost functions, which may differ for different NMF methods. Some NMF variants can also handle negative data, such as convex-NMF, which was the basis of the analysis used in our work (1). Convex-NMF was capable to spot a reduced number of sources confidently recognized as representative from brain tumour/tissue types much better than other NMF variants (2,3). Original sources were extracted from TMZ-treated and control mice, as described in (4), defining prototypic metabolomic patterns related to normal/unaffected brain, GL261 GB tumours actively proliferating and TMZ-treated, responding tumours. The source-based analysis used in our work and other studies (5–7) made use of previously extracted sources in order to analyse new cases and assign one of the three predefined classes to each investigated voxel, thus generating the so-called nosological images.

Nosological images

The nosological images (8) are generated through combination of MRSI approaches with robust machine learning analyses, which take the whole spectral pattern changes into account (i.e. in this case, the 0 - 4.5 ppm spectral vector, see figure S1B). This makes it possible to spot quantitative changes of different metabolites at once, and to detect even subtle variations in metabolomic profile, not easily observed with conventional quantitation. Finally, this approach enabled us to classify the studied tissue into mostly (majority source vote) normal brain tissue, responding or unresponsive tumour.

The labelling of the MRSI data acquired is based on the source extraction technique, which assumes that i) a mixture of the heterogeneous tissue patterns is present in each voxel and that ii) the contribution of individual sources to the final pattern can be calculated. The nosological imaging is generated by estimating the quantitative contribution of each source - paradigmatic spectra - to individual voxels in the MRSI grid, in order to assign them one of the predetermined classes stated above. Thus, the paradigmatic spectrum (source) with the largest contribution to a given voxel is selected as the "winning source", having each source a determined colour. Finally, voxels are assigned the corresponding colours and are represented as nosological maps for each matrix. Green colour is used when the 'GB responding to treatment' source contributes the most, while red is used for 'actively proliferating GB', blue for 'normal brain parenchyma' and black for 'undetermined tissue'. These nosological images can provide a visual representation of MRSI results and be used as an imaging biomarker to determine therapy-caused response.

The most remarkable changes between responding and unresponsive tumours can be summarised. Main metabolites contributing to different patterns of responding and non-responding spectra are mobile lipids 0.9 + macromolecules (MoL, MM, 0.9 ppm), mobile lipids 1.3 + lactate (MoL/Lac, 1.3 ppm), N-acetyl-aspartate and N-acetyl group-containing compounds (NAA and NAc, 2.02 ppm), glutamate + glutamine (Glx, 2.1–2.4 ppm), polyunsaturated fatty acids in mobile lipids (PUFA, 2.8 ppm), total creatine (Cre, 3.03 ppm), choline-containing compounds (Cho, 3.21 ppm), myo-inositol + glycine (Ins + Gly, 3.55 ppm), glutamine + glutamate (Glx, 3.8 ppm, which is also partially contributed by alanine), and lactate (Lac, 1.3 and 4.1 ppm). Figure S1 provides a summary of the steps performed in this type of analysis.

Supplementary Figures

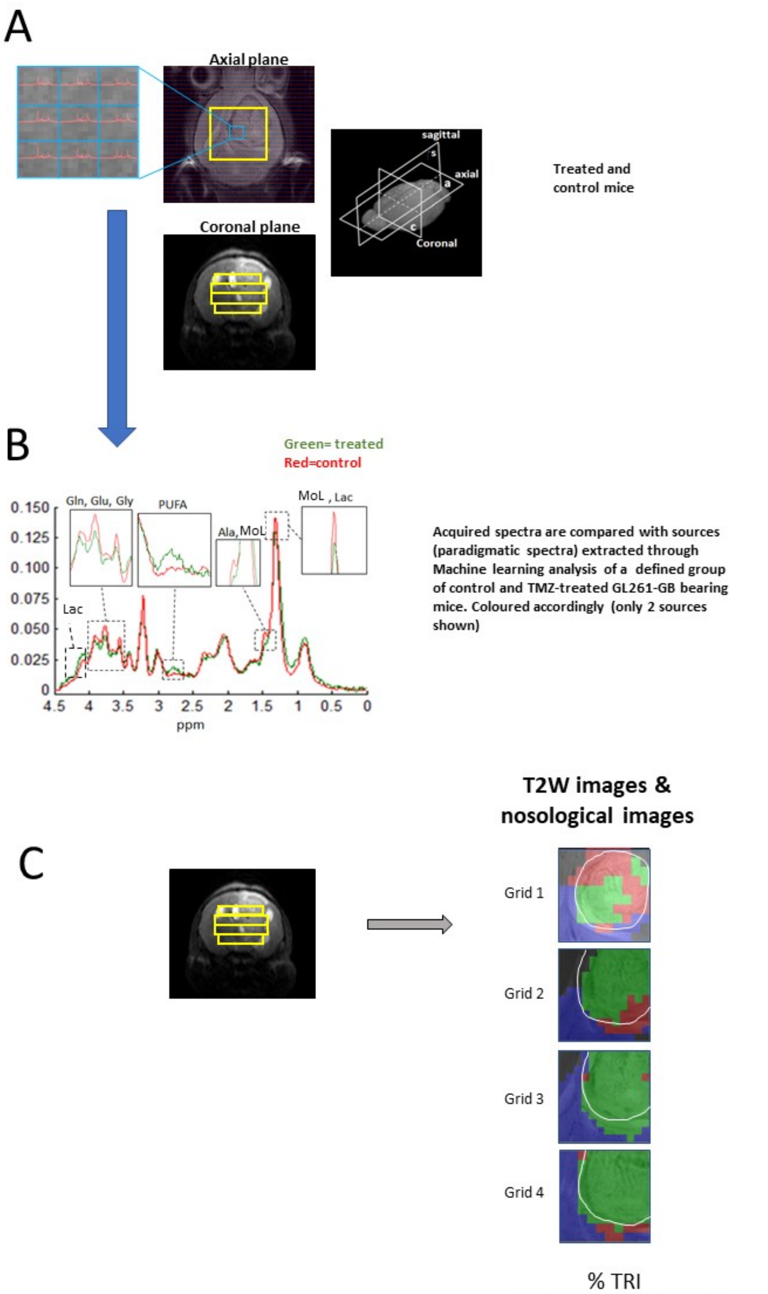

$$TRI = \frac{Tumour\ responding\ pixels}{Total\ tumour\ pixels} x100$$

Figure S1. Summary of steps performed for nosological images calculation mentioned in this work. **A**) MRSI spectra are acquired. Both coronal and axial imaging orientation are shown for better understanding of different anatomical levels studied. Each individual spectrum of the MRSI grid (blue square in A) is analysed as a unique spectral vector. **B**) Spectra are compared with fixed sources (paradigmatic spectra), obtained from a defined group of TMZ-treated and control mice [4]. In fact, 3 sources were acquired but only sources corresponding to control – actively proliferating- and TMZ-treated mice, transiently responding to therapy, are shown, respectively in red and green. Those are the sources relevant to this particular study. Main contributing metabolites are shown in expansion regions. **C**) Spectra, and volume elements, are then coloured according to whether they show more correlation with the control, TMZ-treated or normal brain parenchyma source. If the correlation between the spectrum of a voxel and the sum of the percentages contributed by the sum of the three tissue sources was below a threshold of 50%, this voxel was labelled as 'undecided' and coloured in black [4]. The Tumour Responding Index (TRI) is then calculated as shown in the formula at the bottom, considering spectra (pixels)

identified as responding regarding to the total tumour pixels, taking into account manual drawing over the abnormal section in T2w MRI.

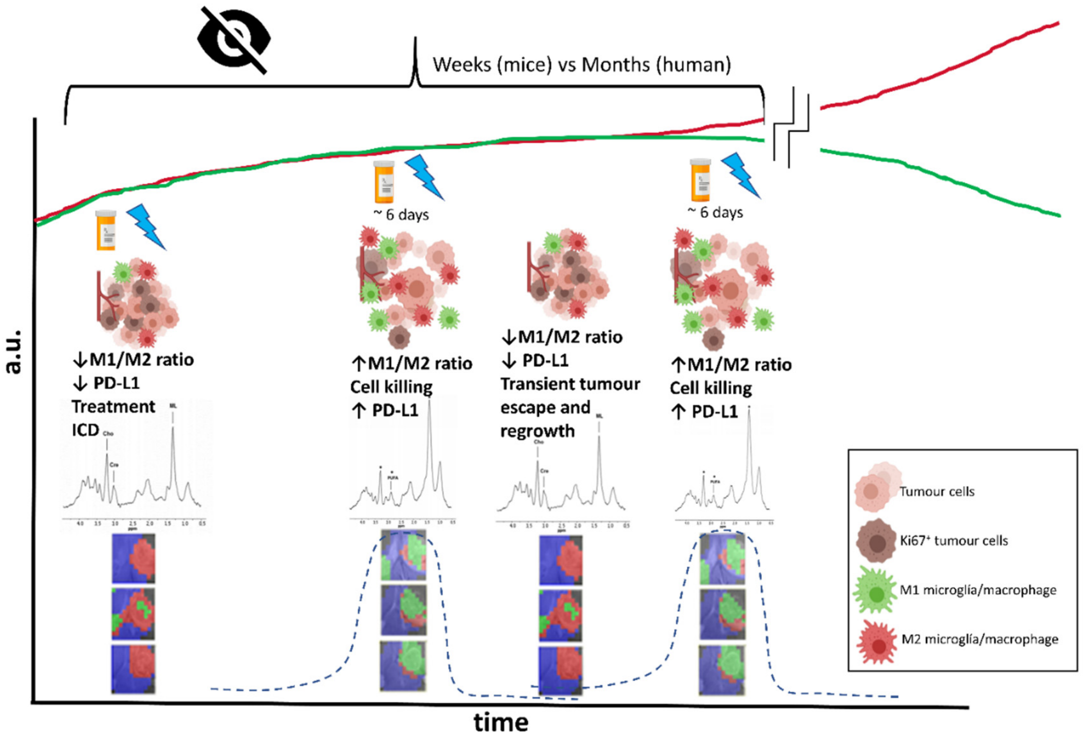

Figure S2. Hypothetical scheme for the rationale behind changes in the nosological images coding for response in MRSI of IMS-TMZ treated GB GL261 bearing mice (see main text and references (5,9) for further details). The oscillation in cellular populations such as Ki67 positive tumour cells or microglia/macrophages, changes in their polarisation status, in surface receptor such as PD-L1 and their subsequent effects, as well as changes in tissue microstructure (giant cells, acellular spaces (5,10)), should all contribute to the MRSI-detected imaging biomarker differences. Those show periodic oscillations that agree with the length of the immune cycle (ca. 6 days) and should be related to events triggered by TMZ therapy. This may be extended (6) to any therapeutic approach eliciting the host immune system. It is also worth noting that these events take place early in the timeline, far before any changes are seen in tumour volumes (red and green lines). Hence detection of such oscillatory changes could provide early indication about immune system-driven response to therapy, while tumour volume changes may be 'blind' to these early local metabolomics changes. At the therapy starting point, GL261 GB tumours display an M2/GAMs ratio which is more than 10-fold higher the M1/GAMs ratio, thus an essentially protumoural microglia/macrophage phenotype (encoded in the red colour over the tumour mass of the nosological images). Launching TMZ therapy may trigger immunogenic cell damage and release/exposure of immunogenic signals (11) which will turn on the cancer immunity cycle and elicit the host immune system (12). In the meanwhile, both M1 and M2 populations infiltrating the tumour increase (see also Figure 2A in [(9) *https://www.mdpi.com/2072-6694/13/11/2663*]): while M1 microglia/macrophages will participate in tumour cell killing, probably after interaction with T-cells (13), and M2 microglia/macrophages are waiting for M1 polarisation. In this respect, M1/M2 ratio changes towards higher values in tumours showing transient response to IMS-TMZ (Figure 3C in (9); *https://www.mdpi.com/2072-6694/13/11/2663*). The point of maximum response spotted by our non-invasive biomarker (i.e., green colour over the GB tumour mass in nosological images) is ca. 6 days after therapy administration, in line with the length of the immune cycle described in (14). At this point, an increase in PD-L1 gene expression is observed (Figure 4A of (9); *https://www.mdpi.com/2072-6694/13/11/2663*), although it could be either due to

expression by the tumour cell population to evade lymphocyte attack, to expression in microglia/macrophages or both together. Since M1 microglia/macrophages are mostly consumed during the antitumour response events, after such interval, the ratio M1/M2 may shift towards the control values, T lymphocytes may be approaching exhaustion and surviving tumour cells may start proliferating again leading to tumour regrowth (day +9, red colour over the tumour image) until the previous therapeutic administration point (at day +6) resets the immune cycle and produces the next response oscillation (day +12).

Supplementary Tables

	GL621 GB	Human GB
**Evolution**	Fast, induced	Slow, spontaneous
**Immune status**	Immunocompetent	Immunocompetent
**Cell differentiation**	Poor	Poor
**Necrosis with pseudopalisade**	Present	Present
**Invasiveness**	Present	Present
**Response to TMZ**	Transient or Total (depends on the therapeutic schedule)	Transient
**Infiltrating immune system cells***	CD3+ T cells, low frequency of Treg lymphocytes, microglia and macrophages	Microglia and macrophages, MDSC cells, CD3+ T T-cells, Treg lymphocytes
**CD133 staining (stem cell like phenotype)**	Positive (depends on cell culture conditions)	Positive
**MHC I expression**	Moderate	low
**MHC II expression**	low	low
**B7-1, B7-2 expression**	low	low
**Mutational load**	High	Low (except in relapsing GB)
**p53 mutation**	Present	Present
**Expression of c-myc**	High	High
**K-ras mutation**	Present	Present
**IDH1 mutation**	Absent (possibility to introduce mutation)	Present in secondary GB (ca. 10%)
**CXCR-4 expression**	Present	Present
**BBB STATUS; enhancement after CA administration**	Compromised since early stage; homogeneous enhancement	Compromised; enhancement could be heterogeneous
**Angiogenesis and hypoxia**	Present	Present
**Increased CD31 expression**	Present	Present
**Activation of PI3K pathway**	Present	Present
**Indoleamine 2,3-deoxygenase (IDO) enzymes**	Present	Present
**MGMT expression**	Described to be relatively weak	Variable, described to correlate with TMZ therapeutic outcome

Table S1: Main similarities/differences between GL261 tumours and human GB tumours. Data gathered from references (6,15–32). **B7-1, B7-2**: costimulatory molecules, involved in the regulation of T-cell signaling. **BBB**: blood brain barrier. **CA**: contrast agent. **CD31**: cluster of differentiation 31 also known as Platelet endothelial cell adhesion molecule (PECAM-1). **CXCR-4**: C-X-C chemokine receptor type 4. **MDSC**: Myeloid-derived suppressor cells. **MHC**: major histocompatibility complex. **TMZ**: Temozolomide. Green lines indicate similarities and red lines indicate differences.
* The immune infiltration is much more complex both in murine and human GB. Comprehensive descriptions can be found in (30,33). Having in mind different aspects, human GB displays a more immunosuppressive profile than GL261.

ppm	Main metabolite assigned	Additional studies supporting findings
**0.9 – 1.0**	Mobile Lipids	(34,35)
**2.8**	PUFAs in Mobile lipids	(34,36,37)
**1.3**	Lactate / saturated fatty acids	(38–40)
**3.55**	Myo-inositol / glycine	(41–43)

Table S2: Metabolites (ppm and tentative assignment) associated with the main changes observed in the metabolomics profile of control and TMZ-treated GL261 GB described by us (4,10)

Supplementary References

1. Ding C, Li T, Jordan MI. Convex and Semi-Nonnegative Matrix Factorizations. *IEEE Trans Pattern Anal Mach Intell* (2010) **32**:45–55. doi:10.1109/TPAMI.2008.277

2. Ortega-Martorell S, Lisboa PJG, Vellido A, Julia-Sape M, Arus C. Non-negative Matrix Factorisation methods for the spectral decomposition of MRS data from human brain tumours. *BMC Bioinformatics* (2012) **13**:38. doi:10.1186/1471-2105-13-38

3. Ortega-Martorell S, Lisboa PJG, Vellido A, Simões R V., Pumarola M, Julià-Sapé M, Arús C. Convex Non-Negative Matrix Factorization for Brain Tumor Delimitation from MRSI Data. *PLoS One* (2012) **7**:e47824. doi:10.1371/journal.pone.0047824

4. Delgado-Goñi T, Ortega-Martorell S, Ciezka M, Olier I, Candiota AP, Julià-Sapé M, Fernández F, Pumarola M, Lisboa PJ, Arús C. MRSI-based molecular imaging of therapy response to temozolomide in preclinical glioblastoma using source analysis. *NMR Biomed* (2016) **29**:732–743. doi:10.1002/nbm.3521

5. Arias-Ramos N, Ferrer-Font L, Lope-Piedrafita S, Mocioiu V, Julià-Sapé M, Pumarola M, Arús C, Candiota AP. Metabolomics of therapy response in preclinical glioblastoma: A multi-slice MRSI-based volumetric analysis for noninvasive assessment of temozolomide treatment. *Metabolites* (2017) **7**:20. doi:10.3390/metabo7020020

6. Wu S, Calero-Pérez P, Villamañan L, Arias-Ramos N, Pumarola M, Ortega-Martorell S, Julià-Sapé M, Arús C, Candiota AP. Anti-tumour immune response in GL261 glioblastoma generated by Temozolomide Immune-Enhancing Metronomic Schedule monitored with MRSI-based nosological images. *NMR Biomed* (2020) **33**:e4229. doi:10.1002/nbm.4229

7. Wu S, Calero-Pérez P, Arús C, Candiota AP. Anti-pd-1 immunotherapy in preclinical gl261 glioblastoma: Influence of therapeutic parameters and non-invasive response biomarker assessment with mrsi-based approaches. *Int J Mol Sci* (2020) **21**:8775. doi:10.3390/ijms21228775

8. De Edelenyi FS, Rubin C, Estève F, Grand S, Décorps M, Lefournier V, Le Bas JF, Rémy C. A new approach for analyzing proton magnetic resonance spectroscopic images of brain tumors: Nosologic images. *Nat Med* (2000) **6**:1287–1289. doi:10.1038/81401

9. Calero-Pérez P, Wu S, Arús C, Candiota AP. Immune system-related changes in preclinical GL261 glioblastoma under TMZ treatment: explaining MRSI-based nosological imaging findings with RT-PCR analyses. *Cancers (Basel)* (2021) **13**:2663. doi:10.3390/cancers13112663

10. Delgado-Goñi T, Julià-Sapé M, Candiota AP, Pumarola M, Arús C. Molecular imaging coupled to pattern recognition distinguishes response to temozolomide in preclinical glioblastoma. *NMR Biomed* (2014) **27**:1333–1345. doi:10.1002/nbm.3194

11. Villamañan L, Martínez-escardó L, Arús C, Yuste VJ, Candiota AP. Successful partnerships: Exploring the potential of immunogenic signals triggered by TMZ, CX-4945, and combined treatment in Gl261 glioblastoma cells. *Int J Mol Sci* (2021) **22**:3453. doi:10.3390/ijms22073453

12. Chen DS, Mellman I. Oncology meets immunology: The cancer-immunity cycle. *Immunity* (2013) **39**:1–10. doi:10.1016/j.immuni.2013.07.012

13. von Roemeling CA, Wang Y, Qie Y, Yuan H, Zhao H, Liu X, Yang Z, Yang M, Deng W, Bruno KA, et al. Therapeutic modulation of phagocytosis in glioblastoma can activate both innate and adaptive antitumour immunity. *Nat Commun* (2020) **11**:1508. doi:10.1038/s41467-020-15129-8

14. Karman J, Ling C, Sandor M, Fabry Z. Initiation of Immune Responses in Brain Is Promoted by Local Dendritic Cells. *J Immunol* (2004) **173**:2353–2361. doi:10.4049/jimmunol.173.4.2353

15. Wouters R, Bevers S, Riva M, De Smet F, Coosemans A. Immunocompetent mouse models in the search for effective immunotherapy in glioblastoma. *Cancers (Basel)* (2021) **13**:1–24. doi:10.3390/cancers13010019

16. Leten C, Struys T, Dresselaers T, Himmelreich U. In vivo and ex vivo assessment of the blood brain barrier integrity in different glioblastoma animal models. *J Neurooncol* (2014) **119**:297–306. doi:10.1007/S11060-014-1514-2

17. Zagzag D, Miller DC, Chiriboga L, Yee H, Newcomb EW. Green fluorescent protein immunohistochemistry as a novel experimental tool for the detection of glioma cell invasion in vivo. *Brain Pathol* (2003) **13**:34–37. doi:10.1111/J.1750-3639.2003.TB00004.X

18. Jacobs VL, Valdes PA, Hickey WF, De Leo JA. Current review of in vivo GBM rodent models: emphasis on the CNS-1 tumour model. *ASN Neuro* (2011) **3**:e00063. doi:10.1042/AN20110014

19. Stevenson CB, Ehtesham M, Mcmillan KM, Gerardo Valadez J, Edgeworth ML, Price RR, Abel TW, Mapara KY, Thompson RC. CXCR4 Expression is Elevated in Glioblastoma Multiforme and Correlates with an Increase in Intensity and Extent of Peritumoral T2-weighted Magnetic Resonance Imaging Signal Abnormalities NIH Public Access. *Neurosurgery* (2008) **63**:560–570. doi:10.1227/01.NEU.0000324896.26088.EF

20. Musumeci G, Castorina A, Magro G, Cardile V, Castorina S, Ribattie D. Enhanced expression of CD31/platelet endothelial cell adhesion molecule 1 (PECAM1) correlates with hypoxia inducible factor-1 alpha (HIF-1oc) in human glioblastoma multiforme. *Exp Cell Res* (2015) **339**:407–416. doi:10.1016/J.YEXCR.2015.09.007

21. Li M, Bolduc AR, Hoda N, Gamble DN, Dolisca S-B, Bolduc AK, Hoang K, Ashley C, Mccall D, Rojiani AM, et al. The indoleamine 2,3-dioxygenase pathway controls complement-dependent enhancement of chemo-radiation therapy against murine glioblastoma. *J Immunother Cancer* (2014) **2**:21. doi:10.1186/2051-1426-2-21

22. Lim M, Xia Y, Bettegowda C, Weller M. Current state of immunotherapy for glioblastoma. *Nat Rev Clin Oncol* (2018) **15**:422–442. doi:10.1038/S41571-018-0003-5

23. Zhu T, Shen Y, Tang Q, Chen L, Gao H, Zhu J. BCNU/PLGA microspheres: a promising strategy for the treatment of gliomas in mice. *Chinese J Cancer Res* (2014) **26**:81. doi:10.3978/J.ISSN.1000-9604.2014.02.01

24. Newcomb EW, Zagzag D. "The Murine GL261 Glioma Experimental Model to Assess Novel Brain Tumor Treatments," in *CNS Cancer -Cancer Drug Discovery and Development* (Humana Press), 227–241. doi:10.1007/978-1-60327-553-8_12

25. Wu A, Oh S, Wiesner SM, Ericson K, Chen L, Hall WA, Champoux PE, Low WC, Ohlfest JR. Persistence of CD133+ cells in human and mouse glioma cell lines: Detailed characterization of GL261 glioma cells with cancer stem cell-like properties. *Stem Cells Dev* (2008) **17**:173–184. doi:10.1089/SCD.2007.0133

26. Haddad AF, Young JS, Amara D, Berger MS, Raleigh DR, Aghi MK, Butowski NA. Mouse models of glioblastoma for the evaluation of novel therapeutic strategies. *Neuro-Oncology Adv* (2021) **3**:vdab100. doi:10.1093/noajnl/vdab100

27. Oh T, Fakurnejad S, Sayegh ET, Clark AJ, Ivan ME, Sun MZ, Safaee M, Bloch O, James CD, Parsa AT. Immunocompetent murine models for the study of glioblastoma immunotherapy. *J Transl Med* (2014) **12**:Article number 107. doi:10.1186/1479-5876-12-107

28. Zagzag D, Salnikow K, Chiriboga L, Yee H, Lan L, Ali MA, Garcia R, Demaria S, Newcomb EW. Downregulation of major histocompatibility complex antigens in invading glioma cells: stealth invasion of the brain. *Lab Invest* (2005) **85**:328–341. doi:10.1038/LABINVEST.3700233

29. Anderson RCE, Anderson DE, Elder JB, Brown MD, Mandigo CE, Parsa AT, Goodman RR, McKhann GM, Sisti MB, Bruce JN. Lack of B7 expression, not human leukocyte antigen expression, facilitates immune evasion by human malignant gliomas. *Neurosurgery* (2007) **60**:1129–1136. doi:10.1227/01.NEU.0000255460.91892.44

30. McKelvey KJ, Hudson AL, Prasanna Kumar R, Wilmott JS, Attrill GH, Long GV, Scolyer RA, Clarke SJ, Wheeler HR, Diakos CI, et al. Temporal and spatial modulation of the tumor and systemic immune response in the murine Gl261 glioma model. *PLoS One* (2020) **15**:e0226444. doi:10.1371/JOURNAL.PONE.0226444

31. Genoud V, Marinari E, Nikolaev SI, Castle JC, Bukur V, Dietrich PY, Okada H, Walker PR. Responsiveness to anti-PD-1 and anti-CTLA-4 immune checkpoint blockade in SB28 and GL261 mouse glioma models. *Oncoimmunology* (2018) **7**:e1501137. doi:10.1080/2162402X.2018.1501137

32. Kayabolen A, Yilmaz E, Bagci-Onder T. IDH Mutations in Glioma: Double-Edged Sword in Clinical Applications? *Biomedicines* (2021) **9**:799. doi:10.3390/BIOMEDICINES9070799

33. Wang J, Wu J, Wang R, Wang J, Chen D, Cao Y, Fu W, Wang W, Li H, Jiao Y, et al. Single-Cell Atlas Reveals Complexity of the Immunosuppressive Microenvironment of Initial and Recurrent Glioblastoma. *Front Immunol* (2020) **11**:835. doi:10.3389/fimmu.2020.00835

34. Hakumäki JM, Poptani H, Sandmair AM, Ylä-Herttuala S, Kauppinen RA. 1H MRS detects polyunsaturated fatty acid accumulation during gene therapy of glioma: Implications for the in vivo detection of apoptosis. *Nat Med* (1999) **5**:1323–1327. doi:10.1038/15279

35. Liimatainen TJ, Erkkilä AT, Valonen P, Vidgren H, Lakso M, Wong G, Gröhn OHJ, Ylä-Herttuala S, Hakumäki JM. 1H MR spectroscopic imaging of phospholipase-mediated membrane lipid release in apoptotic rat glioma in vivo. *Magn Reson Med* (2008) **59**:1232–1238. doi:10.1002/MRM.21607

36. Mirbahai L, Wilson M, Shaw CS, Mcconville C, Malcomson RDG, Kauppinen RA, Peet AC. Lipid biomarkers of glioma cell growth arrest and cell death detected by 1 H magic angle spinning MRS. *NMR Biomed* (2012) **25**:1253–1262. doi:10.1002/NBM.2796

37. Pan X, Wilson M, McConville C, Arvanitis TN, Griffin JL, Kauppinen RA, Peet AC. Increased unsaturation of lipids in cytoplasmic lipid droplets in DAOY cancer cells in response to cisplatin treatment. *Metabolomics* (2013) **9**:722–729. doi:10.1007/S11306-012-0483-8

38. Dastmalchi F, Deleyrolle LP, Karachi A, Mitchell DA, Rahman M. Metabolomics Monitoring of Treatment Response to Brain Tumor Immunotherapy. *Front Oncol* (2021) **11**:691246. doi:10.3389/FONC.2021.691246

39. Lim H, Martínez-Santiesteban F, Jensen MD, Chen A, Wong E, Scholl TJ. Monitoring Early Changes in Tumor Metabolism in Response to Therapy Using Hyperpolarized 13C MRSI in a Preclinical Model of Glioma. *Tomography* (2020) **6**:290. doi:10.18383/J.TOM.2020.00024

40. Hvinden IC, Berg HE, Sachse D, Skaga E, Skottvoll FS, Lundanes E, Sandberg CJ, Vik-Mo EO, Rise F, Wilson SR. Nuclear Magnetic Resonance Spectroscopy to Identify Metabolite Biomarkers of Nonresponsiveness to Targeted Therapy in Glioblastoma Tumor Stem Cells. *J Proteome Res* (2019) **18**:2012–2020. doi:10.1021/ACS.JPROTEOME.8B00801

41. Qu JR, Jiang T, Dai JP, Li HL, Luo JP, Li SW, Al L, Jiang TZ. Proton magnetic resonance spectroscopy predicts radiotherapy response and time-to-progression in high-grade gliomas after surgery. *Chin Med J (Engl)* (2012) **125**:4334–4337. doi:10.3760/CMA.J.ISSN.0366-6999.2012.24.003

42. Wei L, Hong S, Yoon Y, Hwang SN, Park JC, Zhang Z, Olson JJ, Hu XP, Shim H. Early prediction of response to Vorinostat in an orthotopic glioma rat model. *NMR Biomed* (2012) **25**:1104. doi:10.1002/NBM.2776

43. Wibom C, Surowiec I, Mörén L, Bergström P, Johansson M, Antti H, Bergenheim AT. Metabolomic patterns in glioblastoma and changes during radiotherapy: A clinical microdialysis study. *J Proteome Res* (2010) **9**:2909–2919. doi:10.1021/pr901088r
